# Development and Psychometric Testing of Perfectionism Inventory to Assess Perfectionism and Academic Stress in University Students: A Cross-Sectional Multi-Centre Study

**DOI:** 10.3390/ejihpe15060094

**Published:** 2025-05-23

**Authors:** Michela Piredda, Alessio Lo Cascio, Anna Marchetti, Laura Campanozzi, Paolo Pellegrino, Marina Mondo, Giorgia Petrucci, Roberto Latina, Maddalena De Maria, Rosaria Alvaro, Maria Grazia De Marinis

**Affiliations:** 1 Research Unit Nursing Science, Department of Medicine and Surgery, Campus Bio-Medico di Roma University, Via Alvaro Del Portillo, 21, 00128 Rome, Italy; m.demarinis@policlinicocampus.it; 2 Department of Biomedicine and Prevention, University of Rome “Tor Vergata”, Via Montpellier, 1, 00133 Rome, Italy; alessio.locascio@hotmail.it (A.L.C.); rosaria.alvaro@gmail.com (R.A.); 3 Research Unit Nursing in Palliative Care, Department of Medicine and Surgery, Fondazione Policlinico Universitario Campus Bio-Medico, Via Alvaro Del Portillo, 200, 00128 Rome, Italy; a.marchetti@policlinicocampus.it (A.M.); p.pellegrino@policlinicocampus.it (P.P.); 4 Research Unit of Bioethics and Humanities, Campus Bio-Medico di Roma University, Via Alvaro del Portillo, 21, 00128 Rome, Italy; l.campanozzi@unicampus.it; 5 Department of Pedagogy, Psychology and Philosophy, University of Cagliari, Via Is Mirrionis, 1, 09123 Cagliari, Italy; mmondo@unica.it; 6 Research Unit of Orthopaedic and Trauma Surgery, Department of Medicine and Surgery, Università Campus Bio-Medico di Roma, 00128 Rome, Italy; g.petrucci@policlinicocampus.it; 7 Department of Health Promotion, Mother and Child Care, Internal Medicine and Medical Specialties, University of Palermo, Piazza Delle Cliniche, 2, 90127 Palermo, Italy; roberto.latina@unipa.it; 8 Department of Life Health Sciences and Health Professions, Link Campus University, Via Del Casale di San Pio V, 44, 00165 Rome, Italy; maddalena.demaria@outlook.it

**Keywords:** academic stress, confirmatory factor analysis, perfectionism, psychometric testing, surveys and questionnaires, roots, students, family, social relationships

## Abstract

Background: Perfectionism is a growing concern among university students, who face high expectations, demanding workloads, and complex academic tasks. These pressures often lead to stress, negatively impacting performance, well-being, and career trajectories. Existing measures of perfectionism and related stress lack focus on their causes and relevance to students. Methods: This study developed and psychometrically tested an inventory assessing the causes (ROOTS), manifestations (MPS-R), and stress (IPSS-R) related to perfectionism. A multicenter cross-sectional online survey was conducted across multiple Italian universities with 469 students. The ROOTS tool was developed, and the MPS and IPSS were adapted following established guidelines. Content validity was examined, and pilot testing was performed. Confirmatory factor analyses tested three-factor models with a second-order factor for each instrument. Construct validity and reliability were also assessed. Results: The ROOTS, MPS-R, and IPSS-R demonstrated strong structural and construct validity, with acceptable reliability. Significant correlations highlighted the interconnectedness of perfectionism’s causes, manifestations, and stress. Conclusions: The Perfectionism Inventory offers a comprehensive tool for identifying causes, manifestations, and consequences of perfectionism in university students. It can help educators and policymakers develop strategies to mitigate its impact on mental health and academic success. Future research should explore its applicability in other populations.

## 1. Introduction

Perfectionism is an increasing societal concern among young people (Flett & Hewitt, 2020). Many report rising pressure to meet unrealistic standards, contributing to the growth of psychopathology in this group (Curran & Hill, 2019; Collishaw & Sellers, 2020; McManus et al., 2019). This issue is especially relevant in university settings, where students in fields like physics, engineering, and healthcare are expected to think critically and work with precision, fostering perfectionistic tendencies to prevent mistakes (Hiçdurmaz & Aydin, 2017). This phenomenon can begin during undergraduate training, making students susceptible to perfectionism (Lee et al., 2021).

University students face high levels of stress due to academic, social, and financial challenges (Beiter et al., 2015). Key stressors include emotional engagement, high performance expectations, heavy workloads, and time constraints (Pascoe et al., 2020; Regehr et al., 2013). Feelings of inadequacy, often resulting from poor preparation, can further exacerbate stress (Welch, 2023). Balancing part-time jobs or extracurriculars alongside academic demands intensifies strain (Hurst et al., 2013). This stress can lead to poor academic performance and decreased overall well-being, often manifesting as anxiety, depression, and burnout (Bayram & Bilgel, 2008; Bewick et al., 2010; Liu et al., 2019). High stress levels may also contribute to dropout rates, impacting workforce development in various fields (Banaag et al., 2024). Addressing these stressors comprehensively is essential to improve student well-being and academic success.

Perfectionism is a personality trait defined by a drive for flawlessness, setting excessively high performance standards, and tendencies toward severe self-criticism (Frost et al., 1990; Hewitt & Flett, 1991). The construct is commonly examined through two complementary frameworks: a functional model that distinguishes between adaptive and maladaptive forms, and a multidimensional model that identifies distinct sources and directions of perfectionistic tendencies. Adaptive perfectionism is linked with high achievement motivation, conscientiousness, and emotional resilience, whereas maladaptive perfectionism involves self-criticism, fear of failure and psychological distress (Flett et al., 2011; Slade & Owens, 1998; Suh et al., 2017). The latter has been associated with a range of mental health concerns, including eating disorders, social phobia, panic disorder, obsessive–compulsive disorders, anxiety and depression (Enns et al., 2002; Limburg et al., 2017; Patterson et al., 2021; Stairs et al., 2012). These, in turn, are significantly associated with suicidal behaviors, which have become increasingly prevalent among young people in recent years (Apicella et al., 2020; Ferreira et al., 2015; Smith et al., 2018). Additionally, maladaptive perfectionism in students across educational levels has been linked to negative academic outcomes, such as lower satisfaction, procrastination, and study-related anxiety (Filippello et al., 2020; Osenk et al., 2020; K. T. Wang et al., 2018; L. Wang et al., 2018; Yang et al., 2020). In contrast, adaptive perfectionism is linked to time management, efficacy, life satisfaction and positive emotions (Blasberg et al., 2016; Enns et al., 2002; Shih, 2017). Some scholars even refer to this form as “excellencism”, emphasizing its constructive essence (Blasberg et al., 2016; Gaudreau et al., 2022). To better understand the underlying structure of perfectionism, theorists have proposed a multidimensional model that differentiates between three orientations: self-oriented, other-oriented, and socially prescribed perfectionism. Self-oriented perfectionism is intrapersonal, involving a demand for perfection from oneself, striving for unrealistically high standards, and self-critical performance evaluations. Other-oriented perfectionism, as an interpersonal dimension, involves unrealistic expectations and harsh evaluation of others. Socially prescribed perfectionism, also interpersonal, reflects the belief that people who are important in one’s life impose perfectionist standards and critically judge one’s performance (Hewitt et al., 1991; Hewitt & Flett, 1991). Those with a perfectionist personality may demand perfection of themselves, expect it from others, or believe that they are only accepted if they achieve perfection. These two perspectives—the functional distinction (adaptive vs. maladaptive) and the structural typology (self-, other-, socially prescribed)—are not mutually exclusive. Rather, they offer complementary lenses for understanding perfectionism. For example, self-oriented perfectionism may include both adaptive elements (e.g., achievement striving) and maladaptive ones (e.g., self-criticism). In contrast, socially prescribed perfectionism is more consistently linked with maladaptive outcomes, such as relational inferiority and fear of inadequacy, and shows strong associations with anxiety, depression, and suicidal ideation (Limburg et al., 2017; Smith et al., 2018). Other-oriented perfectionism, marked by critical and demanding expectations of others, is associated with interpersonal difficulties and traits like narcissism, hostility, and low altruism (Blasberg et al., 2016; Curran & Hill, 2022; Gaudreau et al., 2022; Hewitt et al., 2017; Stoeber, 2014, 2015). By integrating both frameworks, this study aims to provide a nuanced understanding of how different perfectionism dimensions contribute to psychological functioning in youth and academic contexts.

In a recent reflection on 30 years of research, Flett and Hewitt (2020) underline the global rise of perfectionism as ‘a problem in living’, and suggest further investigation into a key question: why do people feel the need to be perfect? Parental expectations and criticism are recognized as central factors in the development of perfectionism (Frost et al., 1990). According to the Perfectionism Social Disconnection Model (PSDM) proposed by Hewitt et al. (2017), perfectionism may arise from insecure attachment and a persistent self-perception of being defective and unworthy of love, often rooted in adverse or asynchronous parent–child relationships (Ko et al., 2019). Moreover, a strong need to belong has been shown to mediate the relationship between preoccupied attachment and the interpersonal components of perfectionism (Chen et al., 2015). Research on other interpersonal sources of perfectionism indicates that, beyond parental influence, peers and teachers also contributed to socially prescribed perfectionism in university students (Perera & Chang, 2015).

Cross-cultural studies have shown cultural norms significantly influence perfectionism, with Eastern cultures emphasizing social and collectivistic orientation, and Western cultures focusing on individualism and self-orientation (Xie et al., 2008). At a societal level, increasing perfectionism in Western countries is often linked to neoliberal values, which promote market-based competition, economic inequalities, and a reward-driven culture that reinforces individualism, ideals of perfection, and meritocracy, linking personal achievement to self-worth. This environment can lead anxious parents to pass on performance anxieties and pressure to excel academically to young people (Curran & Hill, 2022), reinforcing cultural standards that contribute on socially prescribed perfectionism (Perera & Chang, 2015).

Despite extensive research on perfectionism, no tool specifically measures its causal or associated factors. Understanding the origins and contributing factors of perfectionism is essential for developing interventions to prevent or mitigate its harmful effects on young people. Many tools have been developed to assess perfectionism itself (Flett & Hewitt, 2015). Among the most widely tested and used measures of perfectionism are the Multidimensional Perfectionism Scales (MPS), developed by Frost et al. (1990) and Hewitt et al. (1991). The Frost MPS is a 36-item scale that assesses six domains, including four manifestations and two antecedents of perfectionism, such as parental expectations and criticism (Frost et al., 1990). In contrast, the Hewitt MPS (Hewitt et al., 1991) is a 45-item scale that measures three dimensions of perfectionism: self-oriented (SOP), socially prescribed (SPP) and other-oriented (OOP) perfectionism. Responses are rated on a 7-point Likert scale (ranging from 1 = strongly disagree to 7 = strongly agree), with higher scores indicating more maladaptive perfectionistic attitudes and behaviors. The Child and Adolescent Perfectionism Scale (CAPS; Flett et al., 2016) mirrors the structure of the adult-oriented Hewitt MPS but excludes the other-oriented dimension, which reflects the belief that striving to be perfect is important for others. The Almost Perfect Scale—Revised (APS-R; Slaney et al., 2001) was created with a counseling framework in mind and, unlike the MPSs, defines perfectionism as an internally focused construct, contrasting with models that emphasize external influences. It comprises three dimensions: High Standards (setting self-imposed high expectations), Order (a preference for order and organization), and Discrepancy (the perceived gap between personal standards and actual performance). The APS-R introduced the distinction between “adaptive” and “maladaptive” perfectionism, emphasizing the role of self-evaluative processes such as discrepancy. The Hewitt MPS has been culturally adapted for the Italian population, resulting in a reduced 15-item version that has been psychometrically tested (Lombardo et al., 2022). Based on Lombardo et al.’s findings, a similar abbreviated version could be adapted for university students.

In addition to perfectionism manifestations and causal factors, assessing students’ stress is critical. The Perceived Stress Scale (PSS) is a widely used measure of general perceived stress (S. Cohen et al., 1983), including in university populations (Denovan et al., 2017; Khan et al., 2013). The 10-item, 2-factor Italian version of the PSS (IPSS) has shown validity and reliability with samples of precarious workers (Mondo et al., 2021). While existing academic stress measures, like the 34-item Academic Stress inventory (Lin & Chen, 2009), are comprehensive, they are too lengthy for this study. Therefore, we opted to use the Italian Perceived Stress Scale (IPSS) and expand it with items specific to academic stress.

The aim of this study was to develop and psychometrically test an inventory assessing (1) causal factors of perfectionism; (2) manifestations of perfectionism, and (3) perceived academic stress among university students.

## 2. Materials and Methods

### 2.1. Design

This research was designed as a multicenter cross-sectional observational study. Questionnaire development and psychometric testing followed the methodological steps prescribed by Brancato et al. (2006). To conduct and report this study, we also followed the Strengthening the Reporting of Observational studies in Epidemiology (STROBE) checklist (von Elm et al., 2014).

### 2.2. Instrument Development

#### 2.2.1. Conceptualization and Item Development

A narrative literature review was conducted in PubMed and Google Scholar regarding the topics under study. A panel of experts including two faculty members, a university tutor, one psychologist, one philosopher and one methodologist, developed the three instruments included in the inventory through several meetings held across September and October 2023.

Roots of perfectionism. The literature on perfectionism was carefully reviewed to identify studies linking its manifestation to antecedent or causal factors (Chen et al., 2015; Hewitt et al., 2017; Ko et al., 2019). Established measures, such as the Frost MPS (Frost et al., 1990), were also examined to extract constructs that suggest underlying influences—particularly the domains of ‘parental expectations’ and ‘parental criticism’. Based on this review, a pool of 18 preliminary items was developed to assess perceived influences related to self-worth, unconditional love, acceptance, trust, feelings of fundamental defectiveness, and fear of failure. These factors align with developmental antecedents described in models such as the Perfectionism Social Disconnection Model—PSDM (Hewitt et al., 2017), which links perfectionism to insecure attachment and relational disconnection, and are also consistent with attachment-based explanations for perfectionism’s origins (Ko et al., 2019). The initial structure of the Roots of Perfectionism questionnaire was derived by thematically grouping items into three key domains: Relationships with the family, Relationships with the self, and Social relationships. These dimensions reflect central tenets of contemporary theories of perfectionism, particularly the PSDM and attachment theory. The Relationships with the family dimension captures foundational early interactions—such as parental criticism and conditional affection—that are consistently identified as key contributors to the development of perfectionistic tendencies (Frost et al., 1990; Curran & Hill, 2022). The Relationships with the self domain addresses how these early experiences are internalized and shape one’s self-concept—particularly beliefs related to self-worth, shame sensitivity, and the adoption of rigid personal standards. In contrast, the Social relationships dimension encompasses interpersonal influences beyond the family, such as peer and societal pressures, which are especially relevant to the emergence of socially prescribed perfectionism (Perera & Chang, 2015). This domain also reflects constructs such as the need to belong and fear of social rejection, which have been shown to mediate the relationship between attachment insecurity and interpersonal forms of perfectionism (Chen et al., 2015). Together, these three domains offer a theoretically coherent and developmentally grounded framework for assessing individual perceptions of formative experiences that may contribute to—or buffer against—the development of maladaptive perfectionism. Both positively and negatively worded items were included in the Roots of Perfectionism scale (Roots). Scores for negatively worded items were reversed during the analysis so that higher scores on the Roots indicate a more positive perception of the quality of experiences believed to reduce vulnerability to maladaptive perfectionism.

Perfectionism. The 45-item Hewitt MPS (Hewitt et al., 1991), which investigates the following three dimensions: self-oriented (SOP), socially prescribed (SPP) and other-oriented (OOP) perfectionism, was used. In line with previous studies validating shortened versions, five items of each dimension were selected for use with university students, forming the 15-item Revised MPS (MPS-R) (Lombardo et al., 2022). Items describing positive manifestations were reverse-scored during analysis so that higher scores on each domain of the MPS-R reflect maladaptive perfectionism.

Academic stress. Following a literature review (Bedewy & Gabriel, 2015; Lin & Chen, 2009; Stallman & Hurst, 2016), five additional items specifically targeting academic stress related to perfectionism were added to the original 10-item, two-dimensional IPSS (Mondo et al., 2021). In this study, the original dimensions—negative and positive—were renamed stress and coping, respectively. Scores for positively worded (coping) items were reversed during the analysis so that higher scores on each IPSS-R dimension reflect perceived stress.

#### 2.2.2. Item Revision

The draft of the inventory was then sent by email to the expert panel asking them to reflect on them further, evaluate the instruments’ completeness and item clarity, and provide potential suggestions for improvement. After receiving the panelists’ comments, two items were deleted from the draft of the Roots instrument, which finally included 16 items; 14 items were retained for the MPS-R; and 5 items regarding academic stress were added to the 10-item IPSS, resulting in the 15-item IPSS-R.

#### 2.2.3. Content Validity

The resulting draft inventory was then sent by email to a group of 11 experts who were not involved in the previous phase of instrument development (including a psychologist, a counselor, three faculty members, two PhD students and four Master’s students) to evaluate content validity. They were asked to rate the relevance of each item on a 4-point Likert scale from 1: not relevant to 4: highly relevant. Content Validity Indices (CVI) were thus calculated for each item (I-CVI) and for the whole scale (S-CVI). The I-CVI was calculated with the ratio between the number of experts who rated the item as 3 or 4 and the total number of experts. The cut-off point for excellent I-CVI was 0.78, to adjust for chance agreement among ratings (Polit et al., 2007). The experts rated all the items with I-CVI scores above 0.9 (range 0.91–1.0). The Scale Content Validity Index (S-CVI) was calculated for each scale as the mean of I-CVI scores of the items included (Polit et al., 2007). The S-CVI for Roots was 0.95, for MPS-R was 0.96, and for IPSS-R was 0.96. Therefore, no further items were removed or added.

#### 2.2.4. Pilot Testing

The draft was piloted with 76 undergraduate and Master’s students from one university to identify any potential issue regarding the instrument or its completion. Potential participants were provided with information about the study objectives and asked to provide feedback if any problems arose when completing the questionnaire. Preliminary statistical analyses of pilot data (skewness and kurtosis indices, correlations among items of the same scale, factorability indices such as Kaiser Meyer Olkin and Bartlett’s test of sphericity) were satisfactory. Moreover, participants did not report issues regarding specific items, therefore no further modifications were made to the draft.

#### 2.2.5. Instrument

The final inventory included the 16-item Roots, the 14-item MPS-R, and the 15-item IPSS. Questions regarding participants’ sociodemographic information were also included. An online Microsoft 365 form was created for the administration of the instrument. In this study, the order of item and questionnaire presentation was fixed to ensure consistency and minimize participant confusion. Participants first completed the IPSS-R, followed by the MPS-R, the Roots, and the demographic questions.

#### 2.2.6. Setting and Sampling

Confirmatory factor analyses were planned for each scale as they were developed based on theory following a literature review. Therefore, a sample size of at least 200 subjects was considered adequate, but a higher number was sought to enable testing of known group validity (MacCallum et al., 1999). The inclusion criteria were undergraduate and master students attending Italian universities. Exclusion criteria were students aged < 18 years. Convenience sampling was conducted through an institutional mailing list of several Italian universities and personal contacts of the expert panel.

#### 2.2.7. Data Collection for Final Validation

The link to the online form was sent to potential participants by email between January and July 2024. Participants were provided with information about the study objectives and procedures, and were kindly invited to complete the form and, if possible, share the link with colleagues. Among those who consented to a follow-up assessment, a random subsample of 40 participants was selected for test–retest reliability evaluation. They were asked to complete the instrument on two separate occasions, 10 to 14 days apart, to assess its stability.

#### 2.2.8. Data Analysis

Descriptive analyses were conducted for the sample’s socio-demographic variables and items. Skewness and kurtosis indices were computed to ascertain the normality of item distribution. The mean scores for each factor of the inventory scales were calculated by summing up the item scores and dividing by the number of included items. Since the scales of the inventory were adapted or newly developed based on theory, structural validity or dimensionality was tested by conducting confirmatory factor analyses (CFAs), positing a three-factor model for each scale according to the development theory. The following factors were included: Relationships with the family (7 items), Relationships with the self (6 items), and Social relationships (3 items), for Roots; SOP and SPP (5 items each), and OOP (4 items) for MPS-R; stress (6 items), coping (4 items) and academic stress (5 items) for 15-IPSS. A second-order model was also specified and tested at CFA. Maximum likelihood (ML) was planned to be used as a parameter estimator in MPlus for data approaching normal distribution and robust maximum likelihood (MLr), which accounts for non-normality, for data with skewness and kurtosis indices >|1| (Muthén & Muthén, 2017). According to Hoyle’s recommendations (Hoyle, 1995) and a multifaceted approach to testing model fit (Bentler & Hu, 1998), we used several fit indices, including the root mean square error of approximation (RMSEA); the Comparative Fit Index (CFI); the Tucker and Lewis Index (TLI); and the Standardized Root Mean Square Residual (SRMR). Values of RMSEA ≤ 0.06, RMSEA with 90% confidence intervals ≤ 0.05 to ≤0.08; an RMSEA test of close-fit examining the probability that the approximation error is low *p* > 0.05; CFI/TLI > 0.90; and SRMR ≤ 0.08 (Browne & Cudeck, 1992; Hu & Bentler, 1999) indicated a good fit. The χ^2^ statistics were also calculated and interpreted with the above indices. Factor loadings > |0.30| were considered adequate (Tabachnick & Fidell, 2019).

Known group validity was evaluated to ascertain the construct validity of both Roots and 15-IPSS by posing the hypothesis that (a) scores at Roots will be negatively correlated with perfectionism and perceived stress; (b) scores of the new factor of IPSS-R ‘Academic stress’ will be positively correlated with the other two IPSS-R factors; (c) scores of perceived stress will be positively correlated with perfectionism. The correlations between scores were calculated with Pearson’s product-moment correlation coefficients. Correlations of 0.10–0.3 were considered small, correlations of 0.3–0.49 moderate, and correlations ≥ 0.5 strong (J. Cohen, 1988).

The reliability of each scale was assessed in terms of internal consistency with first- and second-order factors. Specifically, the first-order McDonalds’ Omega coefficient (Revelle & Zinbarg, 2009) factor and factor determinacy coefficients (Muthén & Muthén, 2017) were computed. Since the inventory is composed of scales with multiple factors, and second-order factors were tested, more appropriate model-based coefficients such as the global reliability index for multidimensional scales (Raykov & Marcoulides, 2010) and the Omega H by Zinbarg et al. (2006) were computed for each scale.

To evaluate test–retest reliability, the intraclass correlation coefficients (ICCs) and their 95% confidence intervals (CIs) were computed for the scores of each scale through two-way random effects. Values of ICCs lower than 0.5, between 0.5 and 0.75, between 0.75 and 0.9, and higher than 0.90 indicate poor, moderate, good, and excellent reliability, respectively (Koo & Li, 2016).

Significance was set at *p* < 0.05. The software SPSS v.28.00 (IBM Corp., Armonk, NY, USA) and MPlus v.8.10 (Muthén & Muthén, Los Angeles, CA, USA) were used for statistical and psychometric analyses.

### 2.3. Ethical Considerations

The study was conducted in line with the ethical principles of the Helsinki Declaration (World Medical Association, 2024). The relevant Ethics Committees approved the study prior to commencement (Protocol FPUCBM 001.23(45.22)OSS19 April 2023 and 75.23CET2 cbm, 26 October 2023). Data protection and confidentiality of participant identities were guaranteed in compliance with current data protection regulations. Participants’ consent to study participation and data handling was provided online before filling in and returning the questionnaire.

## 3. Results

### 3.1. Sample Characteristics

Of the 469 students who completed the survey, the majority were female (*n* = 338; 72.1%) and undergraduate (*n* = 338; 72.1%), with a mean age of 25.2 years (SD = 6.36; range: 18–57). Most participants were enrolled in bachelor’s or master’s programs in Nursing (*n* = 159; 33.9%), Engineering (*n* = 48; 10.2%), Medicine (*n* = 37; 7.8%), Psychology (*n* = 30; 6.4%), and Business and Economics (*n* = 29; 6.2%), across various Italian universities. More details on participant characteristics are presented in Table 1. Of the total sample, 38 participants also completed the instrument twice for test–retest analysis.

### 3.2. Structural Validity

The distribution of some items of the inventory did not approach univariate normality, with some skewness and kurtosis indices >|1| (Table 2). Therefore, the CFAs were performed using robust maximum likelihood (MLr) as a parameter estimator.

**Figure 1:**
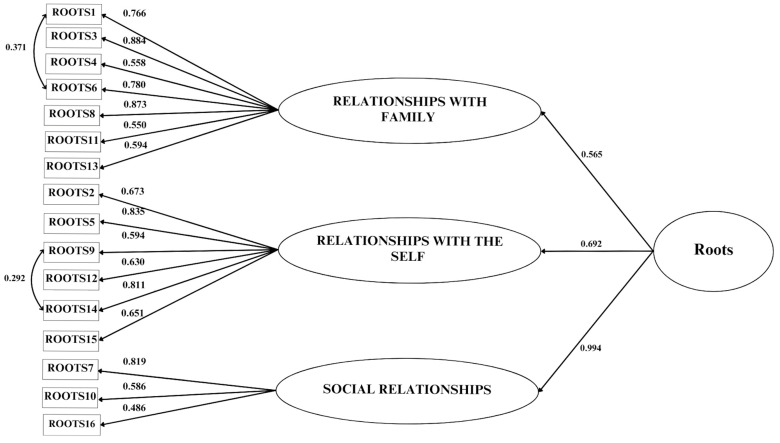
Structural validity of the Roots Scale.

To test the structural validity of Roots, a three-factor confirmatory model was specified: Relationships with family (Family) measured by seven items (#1, #3, #4, #6, #8, #11, #13), Relationships with the self (Self) measured by six items (#2, #5, #9, #12, #14, #15), and Social relationships (Social) measured by three items (#7, #10, #16). The fit indices indicated a misfit: χ^2^(101, *n* = 496) = 288.211, *p* < 0.0001; RMSEA = 0.063 (IC 90% 0.054–0.071), *p* = 0.007; CFI = 0.930; TLI = 0.917; SRMR = 0.056. Following inspection of the modification indices (MI), we specified the covariance between the following items: #1 with #6, and #9 with #14. The fit indices for this model were good: χ^2^(99, *n* = 496) = 228.618, *p* < 0.0001; RMSEA = 0.053 (IC 90% 0.044–0.062), *p* = 0.291; CFI = 0.952; TLI = 0.941; SRMR = 0.050. All the items showed loadings > 0.5 (but item 16 had a loading of 0.49) and *p* values < 0.001 (see Figure 1). Since the three factors were strongly correlated (mean r = 0.55, *p* < 0.001) a second-order model was hypothesized and confirmed, yielding the same fit indices as the first-order model.

**Figure 2:**
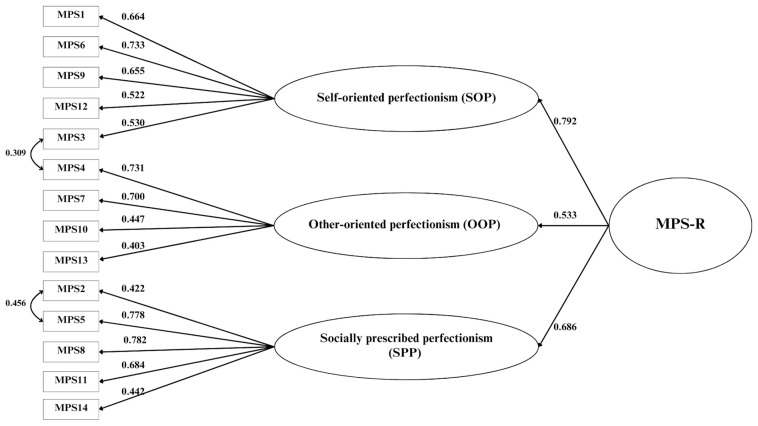
Structural validity of the Multidimensional Perfectionism Scale Revised (MPS-R).

The structural validity of MPS-R was tested through the following three-factor confirmatory model: self-oriented perfectionism (SOP) measured by 5 items (#1, #3, #6, #9, and #12), other-oriented perfectionism (OOP) measured by 4 items (#4, #7, #10, and #13), and socially prescribed perfectionism (SPP) measured by 5 items (#2, #5, #8, #11, and #14). The fit indices indicated a misfit: χ^2^(74, *n* = 469) = 225.014, *p* < 0.0001; RMSEA = 0.066 (IC 90% 0.056–0.076), *p* < 0.004; CFI = 0.900; TLI = 0.877; SRMR = 0.056. Following inspection of the modification indices (MI), we specified the covariance of several items (#2 with #5, and #3 with #4). The fit indices for this model were good: χ^2^(72, *n* = 469) = 150.366, *p* < 0.001; RMSEA = 0.048 (IC 90% 0.037–0.059), *p* = 0.594; CFI = 0.948; TLI = 0.935; SRMR = 0.049. All the items showed loadings > 0.4 and *p* values < 0.001 (see Figure 2). Since the three factors were moderately correlated (mean r = 0.44, *p* < 0.001) a second-order model was also tested and confirmed with the same fit indices as the first-order model.

To test the structural validity of IPSS-R a three-factor confirmatory model was specified: stress measured by six items (#1, #2, #3, #4, #5, and #6), coping measured by four items (#7, #8, #9, and #10), and academic stress measured by five items (#11, #12, #13, #14, #15). The fit indices for this model showed a misfit: χ^2^(87, *n* = 469) = 242.213, *p* < 0.0001; RMSEA = 0.062 (IC 90% 0.053–0.071), *p* < 0.019; CFI = 0.933; TLI = 0.919; SRMR = 0.047. Following inspection of the MI, we specified the covariance of item #1 with #5, and #11 with #12 and #15. The fit for this model improved and was: χ^2^(84, *n* = 469) = 192.799, *p* < 0.001; RMSEA = 0.053 (IC 90% 0.043–0.062), *p* < 0.321; CFI = 0.953; TLI = 0.942; SRMR = 0.042.

**Figure 3:**
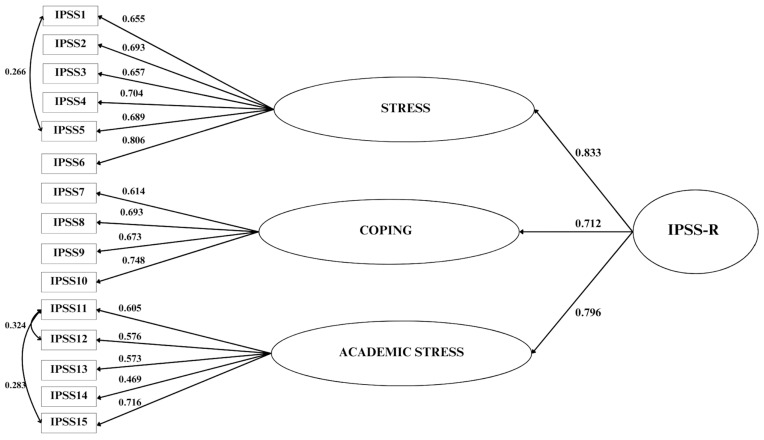
Structural validity of the Italian Perceived Stress Scale Revised (IPSS-R).

All the items showed loadings > 0.5 (but item 14 had a loading of 0.47) and *p*-values < 0.001 (see Figure 3). Since the three factors were strongly correlated (mean r = 0.58, *p* < 0.001) a second-order model was tested and confirmed with the following fit indices: χ^2^(86, *n* = 469) = 245.885, *p* < 0.0001; RMSEA = 0.063 (IC 90% 0.054–0.072), *p* = 0.011; CFI = 0.931; TLI = 0.916; SRMR = 0.061.

### 3.3. Construct Validity

The scores of the Roots factor ‘Social relationships’ were negatively correlated both with MPS-R total scores (r = −0.26, *p* < 0.001) and with those of its factors SPP (r = −0.31, *p* < 0.001) and SOP (r = −0.20, *p* < 0.001). The scores of the Roots factor ‘Relationships with the self’ were negatively correlated with MPS-R total scores (r = −0.48, *p* < 0.001) and with those of its factors: SPP (r = −0.56, *p* < 0.001) and SOP (r = −0.36, *p* < 0.001). The scores of Root factor ‘Relationships with the family’ were negatively correlated with the MPS-R total score (r = −0.16, *p* < 0.001) and with the factor SPP (r = −0.28, *p* < 0.001). Similarly, the scores of all the Roots factors were negatively and significantly correlated with all the IPSS-R factors (see Table 3).

The new IPSS-R factor academic stress was significantly and positively correlated with the other IPSS-R factors Stress (r = 0.50, *p* < 0.001), and Coping (r = 0.26, *p* < 0.001). All the IPSS-R factors were very negatively correlated with Roots factors. Academic stress was also positively and significantly correlated with total MPS-R score (r = 0.44, *p* < 0.001) and with SOP and SPP factors of MPS-R (see Table 3). Therefore, the hypotheses posed to test known group validity were confirmed.

### 3.4. Reliability

The Omega coefficients (omega) for the first-order factors of Roots (Family, Self, and Social) were 0.89, 0.86 and 0.67 and the factor determinacy coefficients were 0.96, 0.94 and 0.85, respectively. The global reliability index for multidimensional scales and the Omega H for the second-order factor were 0.74 and 0.73, respectively. The ICC value for Roots was 0.71 (95%CI: 0.58–0.81, *p* < 0.001) showing moderate test–retest reliability.

The omega values for the first-order factors of MPS-R (SOP, OOP, SPP) were 0.77, 0.67, 0.79, and the factor determinacy coefficients were 0.90, 0.86 and 0.91, respectively. The global reliability index for multidimensional scales and the Omega H for second-order factors were 0.73 and 0.60, respectively. The ICC value for MPS-R was 0.58 (95%CI: 0.42–0.71, *p* < 0.001) indicating poor-to-moderate test–retest reliability.

The omega values for the first-order factors of IPSS-R: Stress, Coping, and Academic Stress were 0.85, 0.78 and 0.79, and the factor determinacy coefficients were 0.93, 0.91 and 0.87, respectively, showing excellent internal consistency. The global reliability index for multidimensional scales and the Omega H for the entire scale were 0.74 and 0.76, respectively (see Table 3). The ICC value for IPSS-R was 0.68 (95%CI: 0.55–0.78, *p* < 0.001) showing moderate test and retest reliability.

## 4. Discussion

This study developed and psychometrically tested a multidimensional Perfectionism Inventory designed specifically for university students incorporating three interrelated components: Roots (causal factors), MPS-R (manifestations), and IPSS-R (stress including academic stress) (See Supplementary File). The inventory responds to longstanding concerns in the literature about the lack of measures that explicitly address the origins of perfectionism rather than merely its expressions or outcomes.

The structural validity of all three instruments was confirmed, and construct validity supported hypothesized relationships between perfectionism causes, manifestations, and perceived stress. As regards the Roots scale, the covariance specified between item #1 (I perceived myself as receiving love from my parents) and item #6 (I trust my parents) is justified by their semantic similarity and the conceptual relationship between love and trust. The covariance between items #9 (I am afraid of failing) and item #14 (When I make a mistake I feel like a failure) reflects their shared focus on the concept of failure. For the MPS-R, the covariance between item #2 (I find it difficult to meet others’ expectations of me) and item #5 (I feel that people are too demanding of me) is theoretically justified, as both items address the perception of external expectations. The covariance between item #3 (One of my goals is to be perfect in everything I do) and #4 (Everything that others do must be of top-notch quality) is attributed to a common underlying construct of perfectionism. However, as these items belong to two distinct dimensions—self-oriented perfectionism (SOP) and other-oriented perfectionism (OOP)—this overlap suggests the need for further psychometric evaluation. Regarding the IPSS-R, the covariances specified between item #1 (Upset because of something that happened unexpectedly) and item #5 (Angered because of things that were outside your control), as well as between item #11 (Pressured by the standards imposed by the institution [school/university]) and items #12 (Strongly pressured by teachers regarding your performance), and item #15 (Your university experience caused you more stress than you could usually handle), are justified by conceptual similarities. Specifically, items #1 and #5 pertain to perceived lack of control over events, while items #11, #12, and #15 relate to academic pressure.

Roots demonstrated strong negative correlations with socially prescribed perfectionism (SPP), the most maladaptive of the MPS-R dimensions, aligning with prior research emphasizing the toxic role of externally imposed expectations (Smith et al., 2018). This supports the theoretical proposition that relational insecurity and conditional regard are central to perfectionism’s maladaptive forms (Chen et al., 2015). The reliability for the second-order factor of MPS-R requires further testing with different samples. Moreover, while some results (e.g., moderate test–retest reliability for MPS-R and IPSS-R) warrant cautious interpretation, these may reflect the state-like fluctuation of stress and perfectionistic behavior over short intervals, rather than limitations in measurement stability. Similarly, adequate internal consistency for the social dimension of Roots and for OOP can be explained by the low number of items included (3 and 4, respectively).

Therefore, they are multidimensional instruments showing good validity, and acceptable reliability. The second order factor confirmed for the three instruments allows for computing more than the scores of single factors, as well as computing an overall score for Roots of perfectionism (Roots), manifestations of perfectionism (MPS-R) and perceived stress (IPSS-R).

Importantly, the Roots measure moves the field beyond symptom description toward an explanatory model of perfectionism. It addresses a crucial gap by capturing early environmental influences, including family dynamics, self-trust, and fear of failure. This aligns with recent calls (Flett & Hewitt, 2020) for greater attention to the “why” of perfectionism—why certain individuals are more vulnerable to developing perfectionistic tendencies under academic and societal pressures.

The most established perfectionism measures, such as the Frost MPS, the Hewitt MPS, the CAPS, and the APS-R, effectively measure what perfectionism looks like, but do not account for why it develops (Sironic & Reeve, 2015). Compared to such prior instruments, which assess key dimensions of perfectionism, the multidimensional Perfectionism Inventory seeks to complement these perspectives by identifying upstream experiential factors that may contribute to (or buffer against) these traits.

The MPS-R component of our inventory addresses self-oriented, socially prescribed and other-oriented perfectionism. The Roots component extends the literature by examining antecedents and potentially modifiable environmental contributors to perfectionism, particularly those related to attachment, conditional self-worth, and early relational experiences, with an emphasis on positive and protective experiences (Chen et al., 2015; Hewitt et al., 2017; Ko et al., 2019). This allows for a more nuanced understanding of how perfectionism develops and persists within students’ psychological and social environments. Moreover, existing tools often treat stress as a downstream consequence but do not measure it in context-specific terms. The academic stress factor introduced in IPSS-R also adds conceptual value. Unlike generic stress measures (e.g., the PSS), this factor captures the specific emotional and cognitive burdens associated with academic life, measuring it as a situationally embedded component. It correlates strongly with SPP and SOP, reinforcing the idea that stress is both a consequence and reinforcing factor of perfectionism in university settings.

In sum, the Perfectionism Inventory represents a conceptual and practical advance over existing instruments. It contributes a causal–expressive–consequential framework, enabling researchers and clinicians to trace the origins of perfectionism, observe its behavioral manifestations, and assess contextual stress responses—a level of conceptual integration not offered by existing tools. This has implications for both research and intervention, supporting efforts to shift from reactive to preventive strategies in student mental health.

### 4.1. Implications for Policy and Practice

This study provides educational institutions with an instrument able to detect not only the manifestations and emotional consequences but also the causes of perfectionism in university students. The inventory should be used regularly to identify, in a timely way, the need for planning support strategies both at the individual and the systemic level, such as the activation of free counseling and tutorial services for students (Vidic & Cherup, 2019). The data collected through the Perfectionism Inventory should be provided to educators so they can pay attention to signs of perfectionism, modulate their teaching accordingly and take action to help students in addressing perfectionism. For instance, educators should stress the value of learning from personal mistakes and using strategies to reduce self-criticism while improving the ability to regulate emotions and thoughts and cognitions in the face of stress, disappointment, and failure (Rice et al., 2016).

During administration of the instrument, respondents should also be asked what strategies they wish were offered by their educational institution, and what strategies already in place they would improve and how. Moreover, given the relational nature of perfectionism and considering the psycho-social and spiritual dimensions of students in an integrated way, it would be advisable to administer the Perfectionism Inventory together with a measure of spiritual well-being. Furthermore, as personal characteristics related with perfectionism such as control, concern and self-confidence are also associated with adaptability, it would also be useful to measure students’ career adaptability (Mondo et al., 2021). The widespread use of the Perfectionism Inventory, for instance at the national or regional level, would provide policymakers and educational institutions with data able to inform the planning of prevention strategies at societal, familial and educational levels.

### 4.2. Strength and Limitations of the Study

The strengths of this study include the development of a novel perfectionism inventory including a measure for the roots of perfectionism, the rigorous methodological steps (Brancato et al., 2006) followed and the robust statistical analyses conducted. The development of Roots, the adaptation of MPS, and the extension of IPSS were grounded in a comprehensive literature review and expert consensus, ensuring the inclusion of the most relevant aspects.

However, the results should be interpreted considering several limitations. The study was conducted nationally with a convenience sample that, despite being multicentric and including university students with diverse characteristics, was skewed toward students in healthcare, economics and law programs. As a result, it may not fully represent the broader university student population. Moreover, the domains of the Roots instrument (family, self, others) and the items representing them require further validation in future research.

### 4.3. Recommendations for Further Research

Future research should test whether the domains of the novel Roots instrument are valid and relevant areas for intervention in the context of perfectionism. Longitudinal studies should assess the stability and predictive utility of these domains, and intervention studies evaluating their responsiveness to therapeutic change. Further studies should investigate whether the findings of this study hold true in different student populations. Developing a similar tool tailored for school students would also be valuable. Future studies should employ randomized and stratified sampling methods to enhance the generalizability of findings across the entire university student population. Additionally, international studies are needed to enable cross-cultural validation of the new instrument.

## 5. Conclusions

This study developed and psychometrically tested the Perfectionism Inventory, which included Roots (assessing the causal factors of perfectionism), MPS-R (measuring manifestations of perfectionism), and IPSS-R (identifying students’ stress), all demonstrating adequate validity and reliability. The Perfectionism Inventory fills an important gap in assessing a growing and concerning phenomenon among young people. Early identification of perfectionism, its underlying causes and its consequences can facilitate timely support strategies to enhance students’ psychosocial well-being, ultimately improving academic success, retention and the quality of future professionals.

## Figures and Tables

**Table 1:** Participants’ characteristics (*n* = 469 university students).

Age (years) Mean, SD (range)	25.19, 6.36 (18–57)	
	***n***	**%**
Gender		
Female	338	72.07
Male	131	27.93
Education		
Bachelor	338	72.07
Master’s degree	131	27.93
Bachelor course year		
1st year	83	17.69
2nd year	176	37.52
3rd year	93	19.82
Master’s course year		
1st year	40	8.52
2nd year	48	10.23
3rd year	29	6.18
Type of university course		
Nursing	159	33.90
Engineering	48	10.23
Medicine	37	7.88
Business and Economics	29	6.18
Psychology	30	6.39
Social Sciences	29	6.18
Sciences and Technology	26	5.54
Literature and Philosophy	24	5.11
Biology	21	4.47
Health Professions	20	4.26
Education	18	3.84
Law	16	3.41
Communication	7	1.49
Others	5	1.07
Region		
Lazio	323	68.87
Sicily	63	13.43
Piedmont	56	11.94
Apulia	12	2.56
Campania	10	2.13
Other	5	1.07

Note: SD = Standard deviation.

**Table 2:** Item descriptive characteristics (*n* = 469 university students).

Item	Mean	SD	Skewness	Kurtosis
Roots				
Roots1. I perceived myself as receiving love from my parents.	5.75	1.49	−1.25	1.07
Roots2. When I make a mistake, I believe that I am at fault.	3.16	1.72	0.62	−0.41
Roots3. I feel accepted by my parents as I am.	5.30	1.73	−0.97	0.10
Roots4. I believe that my opinion is important to my family.	4.93	1.57	−0.64	−0.05
Roots5. No matter what I do, I feel inadequate.	4.36	1.97	−0.19	−1.14
Roots6. I trust my parents.	5.56	1.65	−1.22	0.78
Roots7. I feel accepted by others as I am.	4.52	1.64	−0.43	−0.62
Roots8. I believe that my parents trust me.	5.61	1.49	−1.18	1.07
Roots9. I am afraid of failing.	2.26	1.53	1.34	1.40
Roots10. I feel trusted by others.	5.36	1.23	−0.76	0.89
Roots11. My parents have given me the freedom to choose in important matters.	5.73	1.48	−1.29	1.19
Roots12. I belief I am worthy as a person, regardless of my mistakes.	5.75	1.38	−1.41	2.24
Roots13. My parents are satisfied with my achievements based on my efforts.	5.41	1.48	−1.03	0.77
Roots14. When I make a mistake, I feel like a failure.	3.30	1.94	0.50	−0.83
Roots15. I feel like I don’t have much to be proud of.	4.42	1.94	−0.22	−1.14
Roots16. I feel I can trust others.	3.89	1.57	−0.17	−0.72
MPS-R				
MPS1. When I am working on something, I cannot relax until it is perfect	5.16	1.41	−0.75	0.38
MPS2. I find it difficult to meet others’ expectations of me.	4.21	1.74	−0.18	−0.91
MPS3. One of my goals is to be perfect in everything I do.	4.64	1.72	−0.60	−0.48
MPS4. Everything that others do must be of top-notch quality.	3.22	1.52	0.13	−0.73
MPS5. I feel that people are too demanding of me.	4.39	1.75	−0.34	−0.73
MPS6. It makes me uneasy to see an error in my work.	5.71	1.19	−0.90	0.90
MPS7. I cannot stand to see people close to me make mistakes.	3.13	1.59	0.21	−0.91
MPS8. The people around me expect me to succeed at everything I do	4.61	1.73	−0.46	−0.63
MPS9. I do not have to be the best at whatever I am doing.	3.51	1.78	0.21	−0.94
MPS10. I have high expectations for the people who are important to me.	4.56	1.48	−0.51	0.03
MPS11. My family expects me to be perfect.	3.71	1.87	0.01	−1.08
MPS12. I set very high standards for myself	5.58	1.46	−1.10	0.90
MPS13. The people who matter to me should never let me down	4.74	1.58	−0.48	−0.28
MPS14. Success means that I work even harder to please others	3.06	1.92	0.54	−0.87
IPSS-R				
In the last month, how often have you (been/felt):				
IPSS1. Upset because of something that happened unexpectedly?	1.65	1.06	0.18	−0.49
IPSS2. Unable to control the important things in your life?	1.77	1.08	0.01	−0.59
IPSS3. Nervous and “stressed”?	3.09	0.88	−0.86	0.46
IPSS4. Could not cope with all the things that you had to do?	2.47	1.08	−0.34	−0.49
IPSS5. Angered because of things that were outside your control?	2.38	1.14	−0.26	−0.59
IPSS6. Difficulties were piling up so that you could not overcome them?	1.77	1.22	0.17	−0.81
IPSS7. Confident about your ability to handle your personal problems?	1.59	0.95	0.24	−0.39
IPSS8. Things were going your way?	2.12	0.93	0.11	−0.14
IPSS9. Dealt successfully with irritating life hassles?	1.94	0.90	0.10	−0.01
IPSS10. You were on top of things?	2.16	0.97	0.03	−0.43
IPSS11. Pressured by the standards imposed by the institution (school/university)?	2.73	1.17	−0.64	−0.41
IPSS12. Strongly pressured by teachers regarding your performance?	1.60	1.35	0.36	−1.05
IPSS13. Competition with classmates about grades was very intense?	1.30	1.37	0.69	−0.82
IPSS14. Strongly pressured by your family about your grades?	1.18	1.37	0.78	−0.75
IPSS15. Your university experience caused you more stress than you could usually handle?	2.41	1.26	−0.30	−0.96

Note: SD = Standard Deviation.

**Table 3:** Scores, reliability and correlations of MPS-R, IPSS-R and Roots (*n* = 469 nursing students).

	Mean	SD	ꙍ	ICC	FSD	Fam	Self	Soc	Roots	SOP	OOP	SPP	MPS-R	Stress	Coping	Acad	IPSS-R
Fam	5.47	1.19	0.89		0.96	1											
Self	3.87	1.34	0.86		0.94	0.33 **	1										
Soc	4.59	1.15	0.67		0.85	0.44 **	0.50 **	1									
Roots	4.71	0.97	0.89	0.71	0.84	0.80 **	0.80 **	0.71 **	1								
SOP	4.92	1.09	0.77		0.90	−0.03	−0.36 **	−0.20 **	−0.24 **	1							
OOP	3.91	1.09	0.67		0.86	−0.00	−0.06	−0.03	−0.04	0.28 **	1						
SPP	4.00	1.31	0.79		0.91	−0.28 **	−0.56 **	−0.31 **	−0.50 **	0.43 **	0.25 **	1					
MPS-R	4.30	0.87	0.81	0.58	0.80	−0.16 **	−0.48 **	−0.26 **	−0.39 **	0.78 **	0.61 **	0.81 **	1				
Stress	2.19	0.81	0.85		0.93	−0.20 **	−0.50 **	−0.26 **	−0.42 **	0.23 **	0.02	0.37 **	0.31 **	1			
Coping	1.96	0.73	0.78		0.91	−0.23 **	−0.54 **	−0.38 **	−0.49 **	0.14 **	−0.04	0.33 **	0.23 **	0.56 **	1		
Acad	1.84	0.95	0.79		0.87	−0.18 **	−0.44 **	−0.24 **	−0.38 **	0.28 **	0.10 *	0.53 **	0.44 **	0.50 **	0.26 **	1	
IPSS-R	2.01	0.67	0.88	0.68	0.86	−0.25 **	−0.61 **	−0.35 **	−0.52 **	0.28 **	0.05	0.53 **	0.42 **	0.88 **	0.69 **	0.80 **	1

Note: SD = standard deviation; ꙍ = omega coefficient; ICC = intraclass correlation coefficient; FSD = factor score determinacy coefficient; Soc = social relationships—roots; Fam = relationships with family—Roots; Self = relationships with the self—Roots; SPP = socially prescribed perfectionism (MPS-R); SOP = self-oriented perfectionism—MPS-R; OOP = other-oriented perfectionism—MPS-R; MPS-R = perfectionism; Stress = negative subscale—IPSS-R; Coping = positive subscale—IPSS-R; Acad = academic stress—IPSS-R; * *p* < 0.05; ** *p* < 0.01.

## Data Availability

The data supporting the conclusions of this article will be made available by the authors upon reasonable request.

## Supplementary Materials

The following supporting information can be downloaded at: https://www.mdpi.com/article/10.3390/ejihpe15060094/s1, Inventory of Perfectionism.
