# Supplementary material for: Development and Psychometric Testing of Perfectionism Inventory to Assess Perfectionism and Academic Stress in University Students: A Cross-Sectional Multi-Centre Study
Source: Eur J Investig Health Psychol Educ. 2025 May 23;15(6):94. doi: 10.3390/ejihpe15060094 (PMC12191519; doi:10.3390/ejihpe15060094)
Supplement: Supplementary file 1 [file ejihpe-15-00094-s001.zip › ejihpe-3567994-supplementary.pdf]

INVENTORY OF PERFECTIONISM

(IPSS-R)

Below are listed some personal characteristics. Read each statement and indicate how often the described behavior occurred in the last month on a scale from 0 (never) to 4 (very often).

In the last month, how often have you (been/felt):

	**Never**	**Rarely**	**Occasionally**	**Often**	**Very Often**
Q1 Upset because of something that happened unexpectedly?	0	1	2	3	4
Q2 Unable to control the important things in your life?	0	1	2	3	4
Q3 Nervous and "stressed"?	0	1	2	3	4
Q4 Dealing successfully with day-to-day problems and annoyances?	0	1	2	3	4
Q5 Effectively coping with important changes that were occurring in your life?	0	1	2	3	4
Q6 Confident about your ability to handle your personal problems?	0	1	2	3	4
Q7 Things were going your way?	0	1	2	3	4
Q8 Could not cope with all the things that you had to do?	0	1	2	3	4
Q9 Dealt successfully with irritating life hassles?	0	1	2	3	4
Q10 You were on top of things?	0	1	2	3	4
Q11 Pressured by the standards imposed by the institution (school/ university)?	0	1	2	3	4
Q12. Strongly pressured by teachers regarding your performance?	0	1	2	3	4
Q13. That competition with classmates about grades was very intense?	0	1	2	3	4
Q14. Strongly pressured by your family about your grades?	0	1	2	3	4
Q15. That your university experience caused you more stress than you could usually handle?	0	1	2	3	4

MULTIDIMENSIONAL PERFECTIONISM SCALE REVISED (MPS-R)

Below are listed some personal characteristics. Some of them mention parents or family. In cases where parents/family have been less present, they can be understood as the parental figures who have fulfilled that role (e.g., grandparents, uncles, other significant individuals, etc.). In providing answers, if there are differences between father and mother, choose at your discretion the 'parent' (or significant person) who had the greatest impact.

Read each statement and assess whether you agree or disagree with it and to what extent. If you completely agree, mark the number as 7; if you totally disagree, mark the number as 1; if you feel somewhere in between, mark a number between 1 and 7.

	Disagreement						Agreement
1. When I am working on something, I cannot relax until it is perfect.	1	2	3	4	5	6	7
2. I find it difficult to meet others' expectations of me.	1	2	3	4	5	6	7
3. One of my goals is to be perfect in everything I do.	1	2	3	4	5	6	7
4. Everything that others do must be of top quality.	1	2	3	4	5	6	7
5. I feel that people are too demanding of me.	1	2	3	4	5	6	7
6. It makes me uneasy to see an error in my work.	1	2	3	4	5	6	7
7. I cannot stand to see people close to me make mistakes.	1	2	3	4	5	6	7
8 The people around me expect me to succeed at everything I do.	1	2	3	4	5	6	7
9. I do not have to be the best at whatever I am doing.	1	2	3	4	5	6	7
10. I have high expectations for the people who are important to me.	1	2	3	4	5	6	7
11. My family expects me to be perfect.	1	2	3	4	5	6	7
12. I set very high standards for myself.	1	2	3	4	5	6	7
13. The people who matter to me should never let me down.	1	2	3	4	5	6	7
14. Success means that I work even harder to please others.	1	2	3	4	5	6	7

ROOTS

Below are listed some statements about personality traits. Some of them involve parents or family. In cases where parents/family are not very present, they can be understood as the parental figures who performed that role (e.g., grandparents, aunts, other significant individuals). When providing answers, if there are differences between father and mother, choose at your discretion the 'parent' (or significant person) who had the greatest impact.

Read each statement and evaluate whether you agree or disagree with it and to what extent. If you completely agree, mark the number as 7; if you totally disagree, mark the number as 1; if you feel somewhere in between, mark a number between 1 and 7.

	Disagreement						Agreement
1. I perceived myself as receiving love from my parents.	1	2	3	4	5	6	7
2. When I make a mistake, I believe that I am at fault.	1	2	3	4	5	6	7
3. I feel accepted by my parents as I am.	1	2	3	4	5	6	7
4. I believe that my opinion is important to my family.	1	2	3	4	5	6	7
5. No matter what I do, I feel inadequate.	1	2	3	4	5	6	7
6. I trust my parents.	1	2	3	4	5	6	7
7. I feel accepted by others as I am.	1	2	3	4	5	6	7
8. I believe that my parents trust me.	1	2	3	4	5	6	7
9. I am afraid of failing.	1	2	3	4	5	6	7
10. I feel trusted by others.	1	2	3	4	5	6	7
11. My parents have given me the freedom of choice in important matters.	1	2	3	4	5	6	7
12 I belief I am worthy as a person, regardless of my mistakes.	1	2	3	4	5	6	7
13. My parents are satisfied with my achievements based on my efforts.	1	2	3	4	5	6	7
14. When I make a mistake, I feel like a failure.	1	2	3	4	5	6	7
15. I feel like I don't have much to be proud of.	1	2	3	4	5	6	7
16. I feel I can trust others.	1	2	3	4	5	6	7

Now, please provide some data about you.

**Gender**: Male ☐; Female ☐; **Age:** _____; **Education** ________________________________
**Bachelor course year** _____; **University:** ___________________________; **City:** ____________________

If you wish, you can note any comments or suggestions about the questionnaire topics.

____

____

____

____

____

____

____

____

____

____

The questionnaire is complete. Thank you for your cooperation.
